# Measuring Psychosocial Reactions to COVID-19: The *COVID Reaction Scales* (COVID-RS) as a New Assessment Tool

**DOI:** 10.3389/fpsyg.2020.607064

**Published:** 2020-11-19

**Authors:** Álex Escolà-Gascón, Francesc-Xavier Marín, Jordi Rusiñol, Josep Gallifa

**Affiliations:** School of Psychology, Education and Sport Sciences (FPCEE Blanquerna), Ramon Llull University, Barcelona, Spain

**Keywords:** COVID-19, SARS-CoV-2, coronavirus, post-pandemic, coping styles

## Abstract

Knowing and measuring the psychosocial reactions of people to the coronavirus crisis could be useful for predicting citizen responsibility and psychological well-being in the general population. In this research, we present the *COVID Reaction Scales* (COVID-RS), a new tool that can measure and quantify the psychopathological reactions of the population to the COVID-19 crisis. The sample consisted of 667 subjects. Explorative and confirmative factor analyses were applied to examine the validity and reliability of the COVID-RS. Five dimensions were extracted that predicted 35.08% of the variance of the psychopathological reactions: (1) *disorganized behaviors*, (2) *avoidant behaviors*, (3) *maladaptive information consumption*, (4) *herd behaviors* and (5) *loneliness*. The results indicated that social quarantine induces and increases psychopathological reactions. However, emotional loneliness is reduced for each person with whom the respective subject lives during the quarantine. Finally, we can conclude that the COVID-RS has satisfactory validity and reliability. Measuring dysfunctional reactions to COVID-19 can enable the prediction of citizen responsibility.

## Introduction

Most of the studies that related the coronavirus crisis to mental health focused on determining the psychopathological impact of the social quarantines enacted in Western and Asian countries (see Parmet and Sinha, 2020; Venkatesh and Edirappuli, 2020). In general, during the early phases of the pandemic, numerous publications noted significant increases in levels of anxiety and depression, as well as a growing trend of irrational behaviors in the general population (e.g., Ahorsu et al., 2020; Brooks et al., 2020; Lee, 2020; López and Rodó, 2020; Shanafelt et al., 2020). Once the international social quarantining measures were lifted (i.e., reopening of borders between the countries of the European Union), the social and psychiatric consequences of this crisis became more complex to analyze (e.g., Escolà-Gascón et al., 2020; Frías et al., 2020). The main reason is that there are still no behavioral and psychosocial markers that allow effective decisions to be made to prevent the spread of the coronavirus and safeguard the quality of life of the population. As a demonstration of this problem, many scientific articles that gave solutions regarding how to solve this crisis were retracted (see Yeo-Teh and Tang, 2020).

The results of this study offer new statistically valid and consistent psychometric measures to examine the processes of psychosocial adaptation and dysfunctional management of the general population in the face of this international crisis. Specifically, we offer the development of a new scale that aims to characterize and quantify the psychopathological reactions of the general population in response to the coronavirus crisis. This new scale is called the *COVID Reaction Scales* (COVID-RS).

The consequences of the COVID-19 crisis can be summarized hypothetically in three dimensions (in addition to the medical-health dimension): (1) Changes in social behavior (e.g., Armitage and Nellums, 2020; Bavel et al., 2020); (2) Changes in consumption of information (e.g., Innerarity and Colomina, 2020a,b; Masip et al., 2020); and (3) Socioeconomic changes (e.g., Bonaccorsi et al., 2020; Nicola et al., 2020). All these characteristics can be defined in many ways, but in this research, they will be examined from a psychological and clinical perspective (see De Sousa et al., 2020).

The first dimension refers to the various beliefs or conceptions about lifestyle, socialization behaviors and the quality of mental health of people (see Lau et al., 2005; Chan et al., 2020). For example, Zhang and Ma (2020) reported that more than 50% of the Chinese population felt *panic* and *horror* at the possibility of contracting COVID-19 (included also generalized anxiety). Likewise, symptoms related to *posttraumatic stress* were identified (e.g., Boyraz and Legros, 2020; Horesh and Brown, 2020; Liang et al., 2020). *Irrational behaviors* were also observed, associated with stocking up on food and with dietary changes that many people made during confinement, most notably eating high-calorie foods (see Mattioli et al., 2020). Likewise, a sharp increase in *compulsive buying* of hygienic products (especially toilet paper, which was sold out in most supermarkets) was reported (see Pagano et al., 2020; Zhou et al., 2020). This type of behavior is related to *herd behavior* and the pseudoscientific beliefs that the general population have developed in response to the uncertainty surrounding the COVID-19 pandemic (see Escolà-Gascón et al., 2020). Some authors ask whether these behaviors can be explained by the generalized panic and collective fear that the population has perceived in the face of the coronavirus crisis (see Khan et al., 2020).

The second, referring to changes in information consumption, can be characterized during the first months of the pandemic as (1) *accelerated digitization*. This concept means that communication and social interaction were massively digitized (see Innerarity and Colomina, 2020a). (2) *Disintermediation*. This concept refers to the disappearance of media outlets that facilitated the understanding of technical information (see García-Morales, 2020). (3) *Infodemic*, which is an overabundance of COVID-19-related information (see Innerarity and Colomina, 2020b). According to Andreu-Sánchez and Martín-Pascual (2020), one of the consequences of disintermediation is the indiscriminate appearance of *hoaxes* or *“fake news”* about the coronavirus since many local media acted as filters that prevented *disinformation*. Currently, it is the direct consumer of the information who must filter and screen which news he or she decides to believe and which not (see Aleixandre-Benavent et al., 2020). The problem is that not everyone has sufficient skill and knowledge to effectively screen information (see Pulido et al., 2020a). In fact, Pulido et al. (2020b) observed that fake news is “tweeted” or disseminated more on social networks than scientifically-based information. This can have very negative effects on how the population reacts to the pandemic, which could lead to failed preventive health measures against the advancing virus. For example, Escolà-Gascón et al. (2020) found that pseudoscientific beliefs and positive psychotic symptoms had increased significantly after a social quarantine of 57 days (the study was conducted with a Spanish population; bear in mind that the duration of the quarantine varies according to the legislation and the situation of each country). Determining the social consequences of collective psychosis related to the consumption of information is something that is still in the process of analysis, and more results based on scientific evidence are required to reach a conclusive conclusion (see Van Rheenen et al., 2020).

Third, socioeconomic changes represent the most difficult factor to operationalize in psychological terms. This dimension may be best characterized by the records of the regularization of labor promoted by some governments, such as border closures and the suspension of certain social and leisure activities (i.e., restaurants, hotels, sports centers, etc.), the granting of economic in the United States and deferring tax payments or offering tax relief in the United Kingdom and Spain (see Boletín Oficial del Estado, 2020; Deloitte Insights, 2020; Government UK, 2020; Nicola et al., 2020). Actually, a few years ago, Barbisch et al. (2015) had already reflected on the viability of social quarantine by comparing previous mutations of the SARS virus with *ebolavirus* and pointed out that it could have economic consequences that in the medium or long term would not be sustainable for governments. Regarding people who kept their jobs by teleworking, scientific evidence suggests that *fatigue* and *mental exhaustion* are the main psychological consequences of increased perceived *work stress* (e.g., Tavares et al., 2020).

These three factors are directly related to the psychological well-being of the population and the decisions that each person makes regarding how to react to this crisis (e.g., Escolà-Gascón et al., 2020). In reality, psychological decisions and psychopathological reactions were not variables taken into account in the mathematical models that were developed to predict the epidemiological behavior of coronavirus transmission (see Ivorra et al., 2020). The lack of experimental and valid data concerning the psychopathological impact of this crisis questions the effectiveness of these mathematical models to predict the medical and psychosocial consequences derived from the COVID-19 (see Yeo-Teh and Tang, 2020).

The definition of psychopathological reactions in this study are based on the attachment theory developed by Ainsworth and Bowlby (1991). This theory argues that humans face the daily problems of adult life based on learning and the affective bond developed from childhood. Thus, the concept of “reaction” should be understood in this study as the predominant coping style in each subject, determined by their prior relationships and learning. These styles can be psychopathological when affective relationships are learned and developed in a dysfunctional way. Ainsworth and Bowlby (1991) call this dysfunctional quality insecure attachment. Subjects who have an insecure attachment tend to have a negative view of themselves, self-describe and remain in a state of defensive anxiety on a regular basis (read Camps-Pons et al., 2014). Likewise, insecure attachment can be classified into three coping styles: avoidant, dependent-ambivalent, and disorganized. In this research, we will focus on the avoidant (with anxious and paranoid characteristics) and disorganized (with schizoid and schizotypal characteristics) styles. Avoidant attachment is characterized by the presence of social anxiety, attitudes of distrust in social relationships, and feelings of vulnerability. In contrast, disorganized attachment is characterized by the presence of irrational beliefs, impersonal or cold social relationships, and relentless negative thinking. Therefore, the term “psychopathological reactions” refers to coping styles that meet the characteristics of avoidant and disorganized profiles (e.g., Wang et al., 2020). These coping styles acquire much emphasis when international crises or natural catastrophes occur, so they represent an essential object of study (see Sung et al., 2020; Tian et al., 2020).

Finally, the concept of loneliness or levels of loneliness is defined in this investigation as established by de Jong-Gierveld and Kamphuis (1985). This conception is characterized by understanding loneliness based on two main psychological parameters: the lack of emotional support and the subjective suffering that each individual perceives when they are psychologically alone (see also Trejnowska et al., 2020). More concretely, in a pandemic context, loneliness is also defined as the fear of losing social supports or being physically alone, as well as increased anxiety due to the uncertainty regarding what the individual must personally endure (see Hwang et al., 2020).

## Materials and Methods

### Description of the Sample

A total of 667 participants from the general population participated (30.9% were men and 69.1% were women). All of them were of legal age (mean = 32.46; standard deviation = 10.373). A total of 34.5% resided in the community of Catalonia, 28% in Madrid, 19.8% in Castilla-La Mancha and 17.7% resided in Andalusia. All participants were asked the number of people they had lived with during the 57 days of confinement (mean = 2.07; standard deviation = 1.486). Given that the coronavirus had impacted differently in each of the regions, sociodemographic data were collected concerning the educational level, the presence of psychiatric history, and the economy vs. health dilemma. They were also asked if they had contracted the coronavirus disease. Table 1 classifies the four previous variables according to the autonomous community in which each subject resides.

**TABLE 1:** Percentages and counts of the subjects according to each Spanish community.

Social variables	Categories	CAT	Madrid	CLM	Andalusia	Total sample
Education level	High school	18.7%	15%	27.3%	28%	21%
		(43)	(28)	(36)	(33)	(140)
	Basic vocational training	19.6%	20.3%	22.7%	28.8%	22%
		(45)	(38)	(30)	(34)	(147)
	Advanced vocational training	13.5%	18.7%	23.5%	22.9%	18.6%
		(31)	(35)	(31)	(27)	(124)
	University studies	48.3%	46%	26.5%	20.3%	38.4%
		(111)	(86)	(35)	(24)	(256)
Psychiatric antecedents	Not	59.1%	56.7%	55.3%	58.5%	57.6%
		(136)	(106)	(73)	(69)	(384)
	Yes	29.1%	29.9%	28%	28%	28.9%
		(67)	(56)	(37)	(33)	(193)
	Prefer not to answer.	11.7%	13.4%	16.7%	13.6%	13.5%
		(27)	(25)	(22)	(16)	(90)
Did you get sick of coronavirus?	Yes, with diagnostic tests.	8.3%	18.2%	–	–	7.9%
		(19)	(34)			(53)
	Yes, without diagnostic tests.	22.6%	13.9%	9.8%	5.1%	14.5%
		(52)	(26)	(13)	(6)	(97)
	No, but I have had COVID-19 symptoms.	18.7%	22.5%	9.8%	9.3%	16.3%
		(43)	(42)	(13)	(11)	(109)
	No and I did not have COVID-19 symptoms.	50.4%	45.5%	80.3%	85.6%	61.2%
		(116)	(85)	(106)	(101)	(408)
Do you believe that social confinement was and is a necessary measure to prevent the spread of the virus?	Totally yes	32.2%	45.5%	17.4%	18.6%	30.6%
		(74)	(85)	(23)	(22)	(204)
	In the beginning not, but currently yes.	20%	12.8%	10.6%	5.9%	13.6%
		(46)	(24)	(14)	(7)	(91)
	In the beginning yes, but currently not.	28.7%	28.9%	40.9%	33.9%	32.1%
		(66)	(54)	(54)	(40)	(214)
	Absolutely not	19.1%	12.8%	31.1%	41.5%	23.7%
		(44)	(24)	(41)	(49)	(158)

*In brackets are the observed recounts. CLM, Castilla-La Mancha; CAT, Catalonia.*

The sociodemographic information was obtained in a self-reported manner, and the subjects signed written informed consent as voluntary authorization to participate in this research.

### Instruments Used

#### De Jong-Gierveld Loneliness Scale (DJGLS)

The DJGLS is a questionnaire consisting of 11 items that examine the perceived loneliness of the subject according to the social deprivation theoretical model developed by Peplau and Perlman (1982). The items are statements that express different situations and desires for social contact with other people. All of them were written by de Jong-Gierveld and Kamphuis (1985). The answers are coded as follows: “yes” = 2 points, “more or less” = 1 point and “No” = 0 points. It should be noted that items 1, 2, 4, 7, 8, and 11 must be scored inversely, so that “yes” = 0 points, “more or less” = 1 point and “No” = 2 points. All the answers are added together, and the total result will be the direct score of the perceived levels of loneliness. In this study, the Spanish adaptation was developed by Buz et al. (2014). The validity and reliability of the scores of this scale were excellent in their original version, but the Spanish version showed a better internal consistency index than the initial scale (Cronbach’s alpha = 0.91).

#### COVID Reaction Scales (COVID-RS)

This scale was developed by Álex Escolà-Gascón and aimed to measure the psychopathological reactions and the way each subject copes with the coronavirus crisis. It consists of 31 items expressed in the form of statements. The responses are scored according to the Likert model, which ranges from 0 (which means “completely disagree”) to 4 (which means “totally agree”). The items are grouped into five dimensions contrasted and validated in this report: (1) avoidant behaviors (AB); (2) disorganized behaviors (DB); (3) Maladaptive information consumption (MI); (4) Loneliness (LO); and (5) Herd behavior (HB). The development process of the items and the clinical contents that each scale evaluates are described in the procedures section (see Table 2). The reliability and validity of the COVID-RS were analyzed in this study.

**TABLE 2:** Description of the theoretical framework related to the coping styles and COVID-RS questionnaire development.

Theories used in the COVID-RS	Classification used in the COVID-RS	Clinical profiles and main symptoms	Items	Scales’ denomination
Coping styles (e.g., Ainsworth and Bowlby, 1991)	Avoidant style	(1) Social anxiety	Items 2, 4, 7, 8, 11, 12, and 13.	Avoidant behaviors or AB scale
		(2) Distant mistrust		
		(3) Invulnerability desire		
	Disorganized style	(1) Irrational beliefs	Items 1, 3, 5, 6, 9, 10 14, and 15.	Disorganized Behaviors or DB scale
		(2) Impersonal contact		
		(3) Tachypsychia		
Information consumption (e.g., Pulido et al., 2020b)	Infodemia	(1) Anxiety when there is too much information to consult.	Items 16, 19, and 26.	Maladaptive information consumption or MI scale
		(2) Feeling of blockage and psychic saturation.		
		(3) Feelings of confusion and difficulties in differentiating between reliable and unreliable information.		
	Acceleration	(1) Anxiety and obsession to check the latest news.	Items 21, 25, and 27.	
		(2) Compulsive use of digital news.		
		(3) Dependence to the digital media.		
Need for social supports (e.g., de Jong-Gierveld and Kamphuis, 1985)	Loneliness	(1) Miss someone.	Items 28, 29, 30, and 31.	Loneliness or LO scale
		(2) Having no close friends.		
		(3) Miss the bustle of people		
Panic Behaviors (e.g., Escolà-Gascón et al., 2020)	Herd behaviors	(1) Imitation behaviors.	Items 17, 18, 20, 22, 23, and 24.	Herd behaviors or HB scale
		(2) Food obsession.		
		(3) Need to buy a product until it is exhausted.		
		(4) Mass compulsive shopping.		

### Procedures

This research follows an ex post facto or correlational methodological design. The procedure can be classified into two large blocks: the procedure related to the development of the COVID-RS questionnaire and the procedure related to sampling.

#### Development of COVID Reaction Scales (COVID-RS) Items

The items were written taking into account 4 sources of information: (1) the theories related to coping and attachment styles (see Ainsworth and Bowlby, 1991); (2) the statistical evidence describing the changes in information consumption during the COVID-19 crisis (e.g., Pulido et al., 2020b); (3) the loneliness model proposed by de Jong-Gierveld and Kamphuis (1985); and (4) the empirical evidence regarding the most common pathological behaviors during the first social quarantine (see Escolà-Gascón et al., 2020). Table 2 summarizes the clinical indicators of the COVID-RS to specify more clearly the relationship between each construct and item.

In total, 31 items were written in the form of statements or phrases. All of them were reviewed and approved by the research team of this report. Although coding the responses is the same for all items, the COVID-RS was designed to take into account two application contexts: The first 15 items were written to be answered in the current context, and from a more general perspective, they are written in the present tense. The rest of the items are written in the present perfect because they intend to integrate the psychological consequences and possible metric biases derived from the first mass confinement that was experienced in the European Union (e.g., Brooks et al., 2020). This study tests the validity and reliability of the 31 items of the COVID-RS.

#### Development of Sampling

The sample was obtained through the online application and distribution of the two questionnaires specified in the previous section. Google Forms was used to digitize the items and responses. The massive online application of the tests on social networks and WhatsApp began on July 22 and ended on August 04, 2020. The first raw data matrix obtained was cleaned and because 27 of the participants were minors, these cases were eliminated from the original matrix. There were no blank responses, and no missing values were identified. Once the matrix was refined, 667 final subjects remained, which are the responses analyzed in this report. All participants checked the acceptance box before responding to the scales.

#### Ethics Statement

The Committee of Ethical Guarantees of Ramon Llull University, (Barcelona, Spain) reviewed, favorably evaluated, and approved this research. Likewise, the procedures of this study adhere to the Spanish Government Data Protection Act 15/1999 and the Declaration of Helsinki of 1975, revised in 2013.

### Data Analysis

The data were processed with the JAMOVI open-access statistical program (see The Jamovi Project, 2020). First, an exploratory factor analysis (EFA) was applied. The factors were extracted by parallel analysis and the unweighted least squares method (see Reise et al., 2000). The Promax rotation was applied. From the solution obtained in the EFA, the structural equations were applied adjusting a confirmatory factor analysis (CFA) model. The parameters were estimated using the maximum likelihood method, and the respective fit indices provided by the AMOS program (an extension of SPSS 25 specialized in structural equations) were applied. According to Kline (2013) and Abad et al. (2015) the following adjustment indices and thresholds were used: root mean square error of approximation (RMSEA, threshold ≤0.05); adjusted goodness of fit index (AGFI, threshold ≥0.9); parsimony ratio (PRATIO, threshold ≥0.9); parsimony adjustment to the comparative fit index (PCFI, threshold ≥0.8); comparative fit index (CFI, threshold ≥0.95); Tucker-Lewis coefficient (TLI, threshold ≥0.95); and incremental fit index (IFI, threshold ≥0.95).

Given that this program allows obtaining the Bayes information criterion (BIC), Akaike information criterion (AIC) and consistent Akaike information criterion (CAIC) indices, which indicate the degree of misfit in the model, the Mismatch Reduction Ratio (MRR) was estimated following the deviance expression developed by Pardo and Ruiz (2015):

$$R^{2}=\frac{-2\,L\,L_{0}-\left(-2\,L\,L_{1}\right)}{-2\,L\,L_{0}}\approx \frac{A\,I\,C_{0}-\left(A\,I\,C_{1}\,\right)}{A\,I\,C_{0}\,}$$

where

−2*LL*_0_ is the deviation from the null model,

−2*LL*_1_ is the deviation from the proposed theoretical model,

*MRR* is the Mismatch Reduction Ratio,

*AIC*_*0*_ is the AIC index corresponding to the null model and

*AIC*_*1*_ is the AIC index corresponding to the theoretical model.

The reliability of the COVID-RS was calculated from the internal consistency indices based on Cronbach’s alpha. Given that the items are ordinal variables, the *omega* coefficient by McDonald (1999) is:

$$\omega_{t}=\frac{{\left(\sum \lambda_{\text{j}}\right)}^{2}}{\left[{\left(\sum \lambda_{\text{j}}\right)}^{2}+\sum \left(1-\lambda_{\text{j}}^{2}\right)\right]}=\frac{{\left(\sum \lambda_{\text{j}}\right)}^{2}}{\left[{\left(\sum \lambda_{\text{j}}\right)}^{2}+\left(\sum \psi \right)\right]}$$

where λ_*j*_ is the factor loading of item *j*,

$\lambda_{\text{j}}^{2}$ is the communality of item *j*, and

ψ is the unique variance.

According to Abad et al. (2015), the threshold used to interpret omega coefficient and Cronbach’s alpha coefficient is 0.6. The results of this coefficient above this value indicate acceptable internal consistency values. However, for the BIC, AIC, and CAIC indices, there are no specific thresholds values and for this reason the MRR index is used (check this information in Pardo and Ruiz, 2015).

## Results

### Exploratory Factor Analysis

The EFA of all the items of the COVID-RS is presented in Tables 3, 4.

**TABLE 3:** Exploratory factor analysis.

Items	Extracted factors		Uniqueness
	
	Disorganized behaviors	Avoidant behaviors	
15	0.649		0.621
9	0.645		0.602
3	0.600		0.632
5	0.556		0.667
14	0.556		0.671
10	0.528		0.712
6	0.508		0.697
1	0.471		0.717
11		0.663	0.599
4		0.632	0.607
7		0.599	0.664
8		0.578	0.618
12		0.563	0.647
2		0.558	0.672
13		0.542	0.631
Explained variance (%)	8.67%	8.20%	Total = 35.08%
Average variance extracted	0.564	0.591	–

*Explained variance was taken from the original factorial solution without rotation. Promax rotation was applied (N = 667).*

**TABLE 4:** Exploratory factor analysis.

Items	Extracted factors			Uniqueness
	
	Maladaptive information consumption	Herd behaviors	Loneliness	
16	0.672			0.555
19	0.660			0.572
25	0.648			0.603
27	0.612			0.619
21	0.607			0.594
26	0.548			0.675
20		0.677		0.636
22		0.567		0.665
23		0.551		0.645
18		0.524		0.686
24		0.492		0.701
17		0.480		0.663
29			0.610	0.636
30			0.580	0.654
31			0.553	0.696
28			0.464	0.772
Explained variance (%)	7.78%	6.37%	4.06%	Total = 35.08%
Average variance extracted	0.625	0.549	0.552	–

*Promax rotation was applied (N = 667). Explained variance was taken from the original factorial solution without rotation.*

A total of 5 factors were extracted that together explained 35.08% of the variance of the data. The first factor was composed of items 1, 3, 5, 6, 9, 10, 14, and 15. Taking into account the content of the items (see Table 2), it was called *Disorganized behaviors* (DB). The second consisted of items 2, 4, 7, 8, 11, 12, and 13. The content of the items referred to *Avoidant behaviors* (AB). The third group included items 16, 19, 21, 25, 26, and 27, and called *Maladaptive information consumption* (MI). The fourth grouped items 17, 18, 22, 23, and 24, the content of which indicated that it should be called *Herd behavior* (HB). The last factor had items 28, 29, 30, and 31 and was called *Loneliness* (LO). These factors were used for fitting the confirmatory model presented below.

### Confirmatory Factor Analysis

**FIGURE 1:**
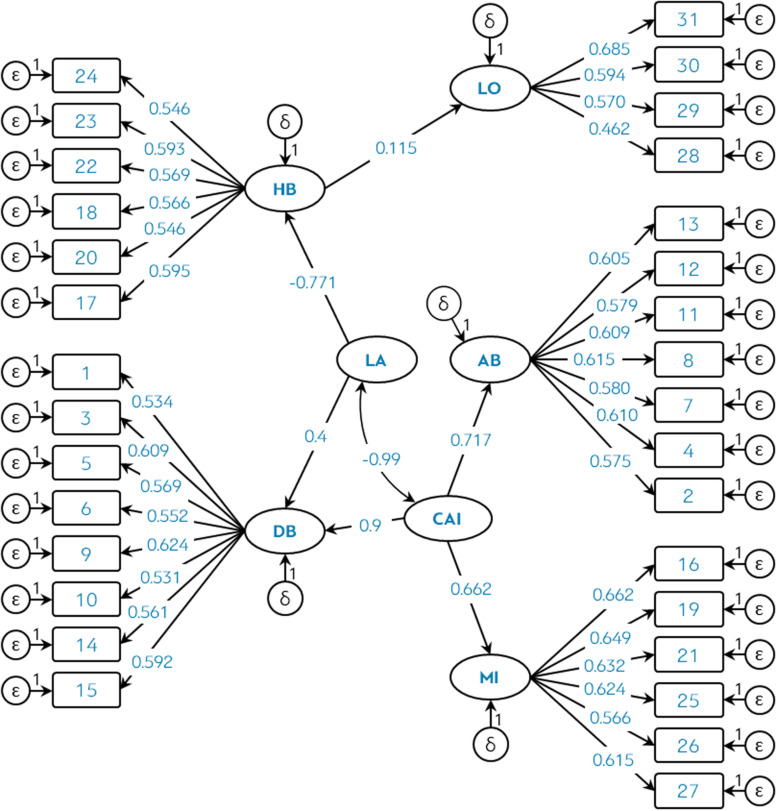
Theoretical model of the COVID-RS scale, showing covariance and standardized regression coefficients. *P*-values were not included for each coefficient because all of them were significant <0.05.

Taking advantage of the results of the EFA, it was then checked whether it was possible to extract new latent variables using a second-order analysis. The content of the items (see Table 2) and the theoretical framework suggested that HB and DB could form a higher-order factor related to *dissociation*. Similarly, AB and DB have in common that their items are related to mistrust (insecure coping style). If we take into account that MI also includes attributes of anxiety, then AB, DB, and MI could form a new higher order factor related to symptoms of anxiety. This logic allowed fitting the confirmatory model of Figure 1.

The latent variables LA (*Lack of awareness*) and CAI (*Coronavirus Anxiety Impact Index*) were defined. Both factors predicted between 43.8 and 57.6% of the variance of the first-order factors extracted in the first EFA.

Table 5 shows the fit indices of the null model (independent) and those of the theoretical model related to the COVID-RS. The table also includes the *Mismatch Reduction Ratio* (MRR).

**TABLE 5:** Model fit indices of the theoretical model (see Figure 1).

Models	Threshold used values (see Kline, 2013; Abad et al., 2015)	Independence model	Theoretical model
χ^2^	–	5019.782	611.099
*p*	–	<0.0001	<0.0001
Normed χ^2^	–	10.795	1.424
RMSEA	<0.05	0.121 (0.118–0.124)	0.025 (0.021–0.030)
AGFI	>0.9	0.414	0.936
PRATIO	>0.9	1	0.923
PCFI	>0.8	∼0	0.886
CFI	>0.95	∼0	0.960
TLI	>0.95	∼0	0.957
IFI	>0.95	∼0	0.960
BIC	–	5221.369	1046.786 (4174.583**) *MRR* = 79.95%
AIC	–	5081.782	745.099 (4336.683**) *MRR* = 85.33%
CAIC	–	5252.369	1113.786 (4138.583**) *MRR* = 78.79%

RMSEA, root mean square error of approximation; AGFI, adjusted goodness of fit index; PRATIO, parsimony ratio; PCFI, parsimony adjustment to the comparative fit index; CFI, comparative fit index; TLI, Tucker–Lewis coefficient; IFI, incremental fit index; BIC, Bayes information criterion; AIC, Akaike information criterion; CAIC, consistent Akaike information criterion; MRR, Mismatch Reduction Ratio estimated using equation [1]. **These values are the differences between independence model and theoretical model.

Although the *Chi Square* statistic has yielded a significance critical level, it should be noted that it is highly sensitive to the sample size, so it becomes inconsistent at the statistical level (see Gorsuch, 1983). Instead, the analysis of the comparative fit indices is recommended, which show values greater than 0.95. Likewise, the RMSEA (*root mean square error of approximation*), AGFI (*adjusted goodness of fit index*), and PRATIO (*parsimony ratio*) indices also showed acceptable and satisfactory values that approve model fit. The estimation of the MRR indicated that the model manages to reduce the misfit between 79 and 85%.

These analyses allow us to conclude that the COVID-RS is a valid questionnaire for examining the psychopathological reactions of the general population to the coronavirus crisis.

### Reliability Analysis

Tables 6–8 present the descriptive statistics associated with the items of both the COVID-RS and of the DJGLS.

**TABLE 6:** Descriptive statistics for all items of the COVID-RS questionnaire.

Items	Mean	Standard deviation	Skewness (error = 0.095)	Kurtosis (error = 0.189)
1	1.48	1.095	0.138	−1.017
2	2.03	1.433	−0.051	−1.332
3	1.52	1.174	0.202	−1.154
4	1.99	1.415	0.017	−1.277
5	1.52	1.178	0.188	−1.11
6	1.52	1.139	0.156	−1.099
7	1.98	1.432	0.022	−1.332
8	1.97	1.392	−0.034	−1.273
9	1.56	1.193	0.173	−1.105
10	1.67	1.188	0.082	−1.076
11	2	1.423	0.004	−1.321
12	1.97	1.412	0.048	−1.308
13	1.95	1.43	0.048	−1.315
14	1.5	1.175	0.257	−1.027
15	1.62	1.205	0.169	−1.055
16	1.94	1.44	0.051	−1.331

**TABLE 7:** Descriptive statistics for all items of the COVID-RS questionnaire.

Items	Mean	Standard deviation	Skewness (error = 0.095)	Kurtosis (error = 0.189)
17	1.6	1.185	0.101	−1.113
18	1.57	1.152	0.009	−1.267
19	2.01	1.408	−0.028	−1.289
20	1.63	1.185	0.065	−1.163
21	1.98	1.357	0.051	−1.181
22	1.54	1.154	0.086	−1.152
23	1.51	1.149	0.162	−1.082
24	1.62	1.152	−0.032	−1.238
25	1.96	1.43	0.017	−1.307
26	2.05	1.343	−0.006	−1.165
27	1.98	1.404	0.004	−1.288
28	2.04	1.399	−0.005	−1.288
29	2.15	1.406	−0.124	−1.26
30	1.97	1.392	0.014	−1.23
31	1.96	1.375	0.071	−1.237

**TABLE 8:** Descriptive statistics for all items of the de Jong Gierveld Loneliness Scale.

Items	Mean	Standard deviation	Skewness (error = 0.095)	Kurtosis (error = 0.189)
1	0.97	0.816	0.061	−1.495
2	1	0.81	−0.003	−1.476
3	0.99	0.809	0.022	−1.472
4	1.01	0.816	−0.022	−1.499
5	1.04	0.804	−0.076	−1.45
6	0.96	0.806	0.071	−1.458
7	0.94	0.802	0.117	−1.435
8	1.02	0.821	−0.031	−1.516
9	1.04	0.822	−0.075	−1.516
10	0.96	0.809	0.079	−1.467
11	0.96	0.837	0.085	−1.568

The descriptive statistics of the scales of both tests and the Cronbach’s alpha and McDonald’s omega reliability coefficients were obtained by summing the responses. This information is presented in Table 9.

**TABLE 9:** Descriptive statistics for all dimensions of the COVID-RS and de Jong Gierveld Loneliness Scale. Reliability coefficients are also included.

Items	Mean	Standard deviation	*Cronbach’s alpha*	*McDonald’s omega*
Disorganized behaviors	12.4	5.992	0.795**	0.795**
Avoidant behaviors	13.89	6.647	0.794**	0.794**
Maladaptive information consumption	11.93	5.88	0.742**	0.742**
Herd behaviors	9.48	4.612	0.794**	0.794**
Loneliness	8.12	3.841	0.632*	0.634*
Lack of awareness	21.88	8.888	0.55	0.6*
Coronavirus anxiety impact index	38.21	14.159	0.642*	0.645*
Total scores of the de *Jong Gierveld Loneliness Scale*	10.88	2.236	0.936***	0.936***

*Acceptable reliability; **Satisfactory reliability; ***Excellent reliability.

In general, the results obtained satisfactorily highlight the reliability of the scores of both the COVID-RS and DJGLS scales. However, the reliability coefficients of the LA factor were the lowest.

### Analysis of Perceived Loneliness

The correlations between the LO, DJGLS scale and, the number of people with whom each subject had lived during the periods of confinement (hereinafter NPPL) were calculated. Table 10 shows the correlation matrix.

**TABLE 10:** Correlation matrix between loneliness scales (LO and DJGLS) and number of people the participant lived with during the social confinement.

variables	Loneliness	DJGLS	NPPL
LO	–		
DJGLS	0.168*	–	
NPPL	−0.426*	−0.087	–

*NPPL, number of people the participant lived with during the social confinement. ^∗^p < 0.0001.*

The *simple linear regression* of the NPPL and LO indicates that, for every person with whom each participant lives, the levels of loneliness are reduced by 1.1 points (within the LO metric, which ranges between 0 and 16). The value 1.1 is the unstandardized regression coefficient or β_1_. The model constant (β_0_) was 10.398. In total, the NPPL variable explains 18% of the reduction in levels of solitude.

## Discussion

The main objective of this study was to facilitate the validity and reliability of new statistical measures concerning the psychopathological reactions of the population amid the COVID-19 crisis. Analyses using structural equations and internal consistency coefficients revealed that the COVID-RS provides valid and reliable scores to measure the psychopathological reactions of the population to this crisis.

### Interpretation and Speculation on the Results

On the one hand, the indices obtained in the factorial analyses (both in their exploratory format and in the model of Figure 1) suggest that the reactions of the population identified in the scientific literature (see Ahorsu et al., 2020; Brooks et al., 2020; Lee, 2020; López and Rodó, 2020; Shanafelt et al., 2020) can be measured validly and reliably in 5 general dimensions: disorganized behaviors (DB), avoidant behaviors (AB), maladaptive information consumption (MI), herd behaviors (HB) and loneliness (LO). This allows for 2 general interpretations:

(1) The presence of the MI dimension supports the results and conclusions obtained in some studies that warn of the social danger of infodemia, disinformation, and the acceleration of digital media. What measures have governments or public organizations applied to control the quality of information about the coronavirus is something that has not been scientifically evaluated (e.g., Escolà-Gascón et al., 2020). However, taking into account the parameters of Figure 1, it cannot be denied that the dysfunctional consumption of COVID-19 information is a psychological reaction that negatively affects the mental health of people. This is because the coronavirus anxiety impact index (CAI) can predict up to 43.82% of dysfunctional information consumption (*R*^2^ ≈ 0.662^2^ = 0.438). Although this measure based on *R*^2^ is an approximate estimate, it is evidence that shows the strength of the relationship between anxiety and the consumption of COVID-19 information. Therefore, it is necessary to provide the general population with digital and psychological resources to promote the correct use of information.
(2) The HB dimension coincides with other studies that warned of the irrational behavior of the population amid the uncertainty related to the COVID-19 crisis (e.g., Pagano et al., 2020; Zhou et al., 2020). Interestingly, the Lack of awareness (LA) index negatively predicted the Herd behaviors (HB) dimension (−0.707). This result is inconsistent with the herd behavior theory since it is precisely the dissociation or disconnection with reality that leads to irrational behaviors that are not logically explained. This negative regression coefficient does not coincide with some studies that positively related herd behaviors with panic behaviors and lack of awareness (e.g., Saglietto et al., 2020). On the one hand, considering the content of the items, this result supports the possibility that HB also measures obsessive-compulsive behaviors, which are positively correlated with cognitive self-consciousness (e.g., Cohen and Calamari, 2004). Then, cognitive self-consciousness would be a mediating variable that could explain the effects of LA on HB. On the other hand, the negative correlation −0.99 between CAI and LA indicates clearly that both indices measure the same construct (anxiety reactions) but from two opposite poles according to the level of consciousness (see Öhman, 2008): LA refers to anxious reactions with low levels of consciousness and CAI is related to anxious reactions with high levels of consciousness. This hypothesis would imply that HB would be positively correlated with CAI. This last logic and classification coincides with the contemporary literature on the psychological evidence identified on coronavirus (e.g., Wang et al., 2020). However, it is recommended in future research to validate the COVID-RS model by including the cognitive self-consciousness variable as a mediator and by estimating an extra parameter that predicts the effects of CAI on HB. Likewise, LA and CAI are hypothetical latent factors. This means that in future studies the concurrent and predictive validity of these two factors should be analyzed with other previously validated anxiety scales.

Finally, the correlation matrix of Table 10 suggests that LO and DJGLS do not measure the same type of loneliness. Like the LA and CAI indices, it is possible that both scales measure different facets of the “loneliness” construct. Analyzing the items of the LO scale, it can be concluded that their contents express the desire for emotional connection and the illusion of sharing leisure time with other people. In contrast, the items of the DJGLS scale focus on the evaluation of social desire but also include 6 items that examine the lack of emotional support. In this sense, it is completely understandable for a person to miss and look forward to being reunited with their loved ones (concept of loneliness measured in LO) and at the same time feel loved and emotionally supported (loneliness evaluated on the DJGLS scale). Therefore, when using the LO scale, it should be taken into account that it is a kind of loneliness based on social and affective desire but not on the lack of psychological support (social deprivation). This argument justifies why the correlation between both scales is so low. Based on these results and if in the future the population should be confined again, the following health/psychological recommendation can be offered: loneliness is less dysfunctional if the subject lives with at least 2 more people. Therefore, it seems advisable to develop confinement situations where people can live with two other people so that deteriorating mental health is not so harmful to people.

### Possible Limitations

The limitations of this research are focused on methodological, theoretical, and sampling aspects.

First, the methodological limitations are mainly found in the reliability coefficients of the LA and CAI indices. Although the *omega* coefficients of both factors reach the minimum acceptance range, they are still low values (see McDonald, 1999). Something similar occurs with the LO scale. How to mathematically manipulate these scales to improve their reliability is something that in psychometric terms is not salvageable with the data of this research. However, based on the negative correlation observed between LO and NPPL, as an alternative to this limitation, it is proposed to include the following mathematical transformation to try to optimize the LO scale scores:

$$L\,O^{\prime }=\frac{\sum_{i=1}^{n}n_{\text{LO}}-W_{\text{NPPL}}}{L\,O_{\text{max}.}}$$

The expression *w*_*NPPL*_ is the number of people with whom the participant lived during confinement. ∑*n*_*LO*_ is the sum of the responses of the items belonging to the LO scale. *LO*_*max.*_ is the maximum score of the LO scale, which in this case would be 16.

Although this formula is intended to be a more effective alternative than the total sum of the responses of the LO items, it should be statistically tested before being used to make clinical decisions. For this, it is proposed to use a new sample (if possible a clinical sample) and to replicate the internal structure of the COVID-RS questionnaire. Likewise, as a complement to this methodological limitation, we should highlight the lack of tests regarding the concurrent, convergent, and discriminant validities. These psychometric properties should be examined in future analyses.

Second, at the conceptual level, it should be noted that the items of the AB and DB scales do not directly measure coping styles; they measure coping styles adapted to the current context of the COVID-19 pandemic. In reality, they reinforce or contextualize the theories of Ainsworth and Bowlby (1991). Therefore, these scales should not be used as direct or explicit measures of coping styles. Along the same lines, there are certain difficulties in interpreting the factors CAI, LA, and LO. Although the results of the structural equations and correlations suggest that CAI represents *fear due to excess activation* or anxiety, LA represents *fear due to the absence of insight*, and LO represents loneliness understood in terms of desires to reunite, new models of structural equations would be necessary to validate its theoretical structure. More specifically and as already suggested, new models should be analyzed to test how the presence of a third factor that groups CAI and LA in the same construct influences the fit and the relationship between these variables.

Finally, the sample used was not recruited using probabilistic procedures, so its representativeness is questionable outside the autonomous communities or regions not included in the analysis. This representativeness is also highly questionable if one takes into account that the subjects come from the general population and not from the clinical-psychiatric population. Thus, new psychometric analyses of the COVID-RS would be necessary in a sample of patients with a formal diagnosis. Likewise, an analysis of the invariance of the COVID-RS scores could be performed including vulnerable groups of the population (i.e., COVID-19 survivors, elderly and medical patients with a risk profile).

### Main Conclusions

The main conclusions that can be deduced from the results and discussion are summarized in the following points:

(1) The COVID Reaction Scales (COVID-RS) is a valid and reliable psychometric test to examine the psychopathological reactions of the population to the coronavirus crisis. The COVID-RS scores can be used as decision criteria to predict how the population will react to government and health measures against the spread of COVID-19. However, before using the COVID-RS for this last purpose, the predictive validity of this scale should be examined. These measures could also be included in the mathematical models that predict the contagion curve for coronavirus.
(2) The psychopathological reactions of the population to the coronavirus crisis can be classified according to the attachment style theory proposed by Ainsworth and Bowlby (1991). Specifically, the structural equations identified two of these styles: avoidant and disorganized. These styles do not provide the population with functional tools for the psychological management of preventative health regulations against coronavirus.
(3) In health and psychological terms, there are reasons and statistical evidence that quarantine states do not harm the mental health and emotional loneliness of the subject when they are in the company of loved ones or family members. Specifically, emotional loneliness is reduced by 1.1 points on the LO scale for each person with whom the respective subject lives during the quarantine.

In general, the COVID-RS scale can be used as a valid and reliable tool for psychological and epidemiological measurement of the reactions of people regarding to their way of coping with the consequences derived from the coronavirus crisis. These measurements can be useful to make effective political and health decisions to confront the COVID-19 crisis successfully.

## Data Availability Statement

The raw data supporting the conclusions of this article will be made available by the authors, without undue reservation, to any qualified researcher.

## Ethics Statement

The studies involving human participants were reviewed and approved by the Committee of Ethical Guarantees of Ramon Llull University, (Barcelona, Spain). Written informed consent for participation was not required for this study in accordance with the national legislation and the institutional requirements.

## Author Contributions

ÁE-G conceived and planned the study, collected the sample, performed the statistical analyses and wrote the manuscript in consultation with F-XM and JR. JG supervised the project. All authors contributed to the article and approved the submitted version.

## Conflict of Interest

The authors declare that the research was conducted in the absence of any commercial or financial relationships that could be construed as a potential conflict of interest.
